# Beating Versus Arrested Heart Technique for Isolated Tricuspid Valve Surgery: A Meta-Analysis of Reconstructed Time-to-Event Data

**DOI:** 10.1177/15569845251351904

**Published:** 2025-07-06

**Authors:** Tulio Caldonazo, Hristo Kirov, Isabel Niedworok, Angelique Runkel, Johannes Fischer, Murat Mukharyamov, Torsten Doenst

**Affiliations:** 1 Department of Cardiothoracic Surgery, Friedrich-Schiller-University Jena, University Hospital Jena, Germany

**Keywords:** beating heart, arrested heart, tricuspid valve surgery

## Abstract

**Objective:**

Isolated tricuspid valve (TV) surgery remains underused despite guideline recommendations. This underuse may be related to perceived high risk in comorbid patients but also to high reported needs for postoperative permanent pacemaker implantation (PPI). It is conceivable that PPI can be prevented by operating on the beating heart (BH). We conducted a systematic review and meta-analysis assessing the influence of BH versus arrested heart (AH) technique on short-term and long-term outcomes after isolated TV surgery with a specific focus on PPI requirements.

**Methods:**

Three databases were assessed. The primary outcome was the rate of postoperative PPI. Secondary endpoints included short-term and long-term survival, cardiopulmonary bypass (CPB) and procedural duration, intensive care unit (ICU) and hospital stay, and postoperative stroke incidence. Hazard ratios, odds ratios, and 95% confidence intervals were calculated. A pooled Kaplan–Meier survival curve after reconstruction analysis was generated for the endpoint of long-term survival. Random-effects models were used.

**Results:**

A total of 1,157 studies were identified. Six observational studies from different countries were included in the analysis. The cohorts receiving either BH or AH technique for isolated TV surgery showed no significant differences in the rate of PPI (range: 6.3% to 18.2%) or any secondary outcomes, including short-term and long-term survival, CPB and procedural duration, ICU and hospital stay, or stroke incidence.

**Conclusions:**

Our meta-analysis suggests that performing TV surgery on the BH is not likely to be associated with a reduced risk of postoperative PPI or with different incidences of major clinical endpoints.

## Graphical abstract

**Visual abstract:**
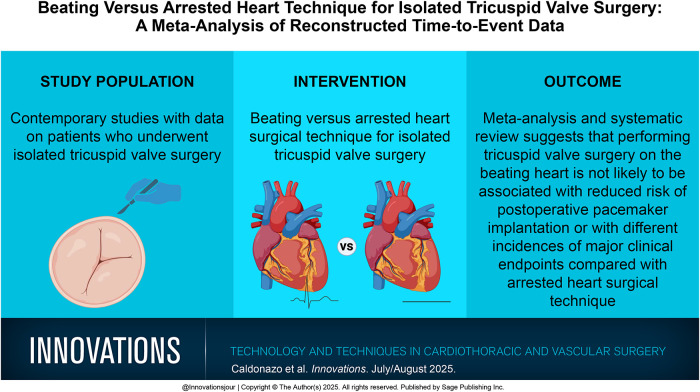

**Central Message**

In this systematic review and meta-analysis, beating heart tricuspid valve surgery was not found to significantly affect the likelihood of postoperative pacemaker implantation or alter the rates of major clinical outcomes compared with arrested heart surgery.

## Introduction

According to the European guidelines, isolated tricuspid valve (TV) surgery should be performed in cases of severe primary or secondary tricuspid regurgitation (TR) that are symptomatic and/or associated with a dilated right ventricle.^1^ Observational studies show that moderate to severe TR occurs with an incidence of up to 3.2% and 4.4% in the male and female elderly population, respectively.^2^

Considering additional etiologies such as right-sided endocarditis and stenosis due to rheumatic disease, alongside others,^3^ these findings may extend to a large number of patients worldwide who would be guideline-conforming candidates for TV surgery. However, in clinical practice, isolated operations on the TV remain underused and are often initiated too late,^4–6^ even though it is known that inadequate treatment might lead to right ventricular damage and peripheral organ failure.^7^ Reasons for this disregard of guidelines in daily practice might be the gaps in randomized evidence^1^ as well as high perioperative mortality and permanent pacemaker implantation (PPI) rates that have been reported in the literature.^8,9^ Although operative mortality is mainly influenced by patient comorbidities,^10^ differences in surgical technique may be responsible for the high PPI rates. Specifically, performing tricuspid repair on the beating heart (BH) has the potential to prevent PPI requirement, because a surgically induced atrioventricular (AV) block may intraoperatively be detected and consequently avoided.^11^

Therefore, the European Society of Cardiology states that BH surgery (even for first-time operations) may prevent AV block and reduce ischemic time, but the organization does not promote official recommendations,^1,12^ probably for the lack of convincing evidence. We performed a systematic review and meta-analysis comparing short-term and long-term outcomes of patients undergoing isolated TV surgery with BH versus arrested heart (AH) technique, with a focus on the need for postoperative PPI.

## Methods

Ethical approval of this analysis was not required as no human or animal subjects were involved. This review was registered with the National Institute for Health Research International Registry of Systematic Reviews (PROSPERO, CRD42024518304).

### Search Strategy

We performed a comprehensive literature search to identify contemporary studies reporting short-term and long-term outcomes of BH and AH technique in patients receiving isolated TV surgery. Searches were run in October 2023 and included the following databases: Ovid MEDLINE, Web of Science, and The Cochrane Library. The search strategy for Ovid MEDLINE is available in Supplemental Table 1.

### Study Selection

The study selection followed the Preferred Reporting Items for Systematic Reviews and Meta-Analyses (PRISMA) strategy. After de-duplication, records were screened by 2 independent reviewers (T.C. and I.N.). Any discrepancies and disagreements were resolved by a third author (H.K.). Titles and abstracts were reviewed against predefined inclusion and exclusion criteria.

### Eligibility Criteria

Studies were considered for inclusion if they reported a direct comparison of outcomes between populations undergoing isolated TV repair or replacement using either the BH or AH technique and were written in English. To conduct this meta-analysis, observational retrospective and prospective as well as randomized controlled trials were considered for further synthesis.

Exclusion criteria were non-English-language publications, studies lacking outcomes of interest, conference abstracts and proceedings, case reports, and noncomparative study designs. All studies reporting outcomes after procedures different than cardiac surgery (i.e., interventional TV repair or other transfemoral invasive cardiological procedures) were excluded. The full text was pulled for a second round of eligibility screening. References for the selected articles were also reviewed for relevant studies not captured by the original search. The quality of the included studies was assessed using the Newcastle–Ottawa Scale (Supplemental Table 2).

Two reviewers (T.C. and I.N.) independently performed data extraction. The accuracy was verified by a third author (H.K.). The extracted variables included study characteristics (publication year, country, sample size, study design, enrollment start and end dates, follow-up, presence or absence from population adjustment, and reported outcomes) as well as preoperative and perioperative patient characteristics (age, sex, mean left ventricular ejection fraction [LVEF], hypertension [HTN], diabetes mellitus [DM], chronic obstructive pulmonary disease [COPD], atrial fibrillation [AF], prior cardiac surgery, preexisting pacemaker, ascites, chronic renal failure, operative procedure for endocarditis, moderate/severe TR, TV repair, median sternotomy, urgent/emergent surgery). No automation tools were used in the study selection or data collection processes.

### Outcomes

The primary outcome was the incidence of PPI. Secondary outcomes were short-term and long-term survival, procedural duration, cardiopulmonary bypass (CPB) duration, intensive care unit (ICU) and hospital length of stay (LOS), as well as perioperative incidence of stroke. Short-term was defined as 30-day or in-hospital outcomes.

### Statistical Analysis

We conducted a meta-analysis to compare the outcomes of BH versus AH technique in isolated TV surgery. Odds ratios (ORs), hazard ratios (HRs), 95% confidence intervals (CIs), and *P* values were calculated for the primary and secondary outcomes. An OR greater than 1 indicated that the outcome was more frequently present in the BH arm. Continuous variables were analyzed using standardized mean difference (SMD) and 95% CI. An SMD greater than 0 corresponded to larger values in the BH arm. The results were displayed in forest plots. The alpha error was set to 0.05. For the secondary outcome of long-term survival, we used a 2-stage meta-analysis as well as a time-to-event data strategy.^13,14^ Inherent clinical heterogeneity between the studies was balanced via the implementation of a random effects models. Between-study statistical heterogeneity was assessed with the Cochran Q statistic and by estimating I^2^. High heterogeneity was confirmed with a significance level of *P* < 0.10 and an I^2^ of at least 50%. Leave-one-out sensitivity analyses were also performed for the outcome of long-term survival.

### Reconstruction of Individual Patient Survival Data

We used the methods described by Wei et al. to reconstruct individual patient data (IPD) from the Kaplan–Meier curves of all eligible studies for long-term survival.^13,14^ Raster and vector images of the Kaplan–Meier survival curves were preprocessed and digitized, so that the values reflecting specific time points with their corresponding survival/mortality information could be extracted. Where additional information was available (e.g., number-at-risk tables or total number of events), it was used to further calibrate the accuracy of the time to events. To confirm the quality of the timing of failure events captured, we thoroughly checked the consistency with the reported survival or morality data provided in the original publications.

### Meta-Analysis of Reconstructed Data

The Kaplan–Meier method was used to calculate the overall survival. The Cox proportional hazards regression model was used to assess between-group differences. For these Cox models, the proportional hazards assumption was verified by plotting scaled Schoenfeld residuals, log–log survival plots, and predicted versus observed survival functions. We plotted the survival curve using the Kaplan–Meier product limit method and calculated the HRs and 95% CIs for each group.

An HR greater than 1 indicated that the outcome was more frequently present in the BH arm. Inherent clinical heterogeneity between the studies was balanced via the implementation of a random-effects model. All statistical analyses were performed using R (version 4.3.1, R Project for Statistical Computing, Vienna, Austria) within RStudio (Posit, Boston, MA, USA) and STATA IC17.0 (StataCorp LLC, College Station, TX, USA).

## Results

### Study Characteristics

**Fig. 1.**
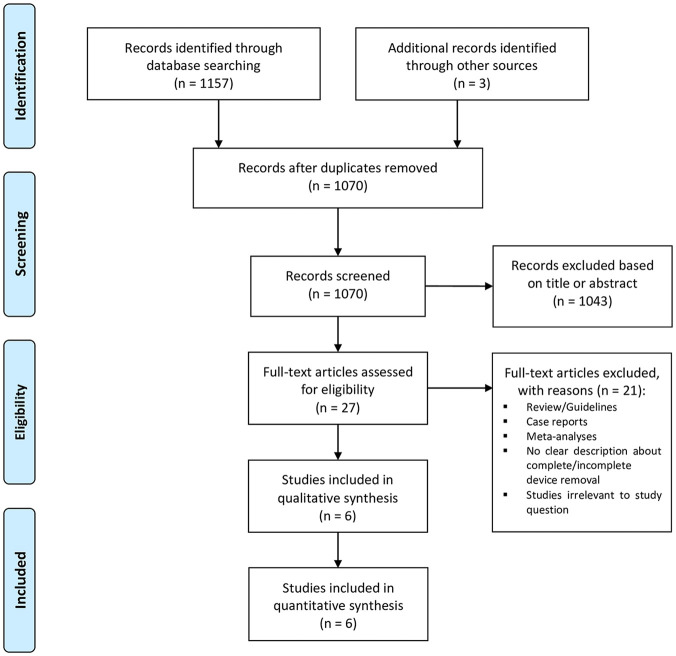
Preferred Reporting Items for Systematic Reviews and Meta-Analyses (PRISMA) flow diagram.

A total of 1,157 studies were retrieved from the systematic search, of which 6 met the criteria for inclusion in the final analysis. Figure 1 shows the PRISMA flowchart for study selection. Included studies were published between 2012 and 2022. All studies were observational cohorts. Two originated from Germany and 1 each from Austria, Turkey, France, and Iran. Table 1 shows the details of the included studies. A total of 767 patients were included in the final analysis. The number of patients in each study ranged from 29 to 406.

**Table 1.** Summary of Included Studies.

Author	Publication year	Country	Patients	Study design	Mean follow-up	Enrollment dates	Population adjustment	Intervention	Reported outcomes
Bigdelu et al.^15^	2022	Iran	53 21 BH, 32 AH	Multicentral, retrospective, nonrandomized	NR (in-hospital data)	2011–2018	Unadjusted	Isolated TV surgery (repair and replacement)	Mortality, CPB duration, procedural duration, ICU stay, hospital LOS
Russo et al.^16^	2021	Austria	406 153 BH, 253 AH	Multicentral, retrospective, nonrandomized	21 months (range 1–131)	2008–2019	Propensity-score matching	Isolated TV surgery (repair and replacement)	PPI, mortality, hospital LOS
Flagiello et al.^17^	2021	France	82 47 BH, 35 AH	Unicentral, retrospective, nonrandomized	51.2 ± 37.1 months	2007–2017	Propensity-score matching	Isolated TV surgery (repair and replacement)	PPI, mortality, CPB duration, ICU stay, hospital LOS
Atılgan and Demirdaş^18^	2020	Turkey	29 16 BH, 13 AH	Unicentral, retrospective, nonrandomized	NR (in-hospital data)	2006–2012	Unadjusted	Isolated TV replacement	Mortality, CPB duration, ICU stay, hospital LOS
Baraki et al.^19^	2015	Germany	92 48 BH, 44 AH	Unicentral, retrospective, nonrandomized	4.2 ± 4.0 years BH, 5.5 ± 4.3 years AH	1996–2011	Unadjusted	Isolated TV surgery (repair and replacement)	PPI, mortality, CPB duration, procedural duration, ICU stay, hospital LOS
Pfannmüller et al.^11^	2012	Germany	105 63 BH, 42 AH	Unicentral, retrospective, nonrandomized	32.0 ± 32.6 months	1997–2010	Unadjusted	Isolated TV surgery (repair and replacement)	PPI, mortality, CPB duration, procedural duration, perioperative complications

Abbreviations: AH, arrested heart; BH, beating heart; CPB, cardiopulmonary bypass; ICU, intensive care unit; LOS, length of stay; NR, not reported; PPI, permanent pacemaker implantation; TV, tricuspid valve.

### Patient Characteristics

Supplemental Table 3 and Supplemental Table 4 summarize the preoperative and perioperative data of the patient population in each study. The mean patient age ranged from 49.5 to 65.2 years. The percentage of female patients ranged from 25.0% to 76.2%. The mean LVEF ranged from 38.1% to 61.2%. The prevalence of HTN ranged from 31.4% to 63.8%. The prevalence of DM ranged from 2.3% to 37.5%. The prevalence of COPD ranged from 4.5% to 19.0%. The prevalence of AF ranged from 14.3% to 76.2%. Between 22.7% and 84.4% of the patients had undergone previous cardiac surgery. Only 2 studies reported the percentage of preexisting pacemaker, ascites, and chronic renal failure, which ranged from 14.9% to 42.9%, 9.1% to 57.1%, and 13.6% to 43.8%, respectively.

In 12.8% to 57.1% of cases, endocarditis was the indication for TV surgery. Between 48.6% and 95.3% of patients presented with moderate to severe TR as an indication for surgery. TV repair was performed in 0% to 81.0% of cases. The percentage of median sternotomy for TV surgery ranged from 19.0% to 100.0%. Between 4.5% and 22.9% of TV operations were performed in an urgent/emergent setting.

### Primary Outcome

**Fig. 2.**
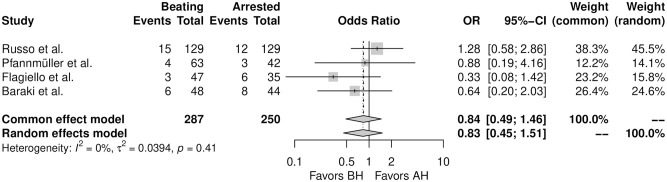
Forest plot for permanent pacemaker implantation. AH, arrested heart; BH, beating heart; CI, confidence interval; OR, odds ratio.

Figure 2 shows the forest plot for PPI. There was no significant difference between the 2 strategies in the incidence of PPI (OR = 0.83, 95% CI: 0.45 to 1.51, *P* = 0.54). Table 2 shows the PPI rates from the individual studies regarding the surgical strategy. The PPI rates ranged from 6.3% to 12.5% in the BH group and from 7.1% to 18.2% in the AH group. The leave-one-out analysis confirmed the robustness of the analysis (Supplemental Fig. 1). Supplemental Figure 2 shows the funnel plot for the primary outcome.

**Table 2.** Individual Studies Postoperative Permanent Pacemaker Implantation Rates.

Study	Beating heart	Arrested heart
Russo et al.^16^	11.6%	9.3%
Pfannmüller et al.^11^	6.3%	7.1%
Flagiello et al.^17^	6.4%	17.1%
Baraki et al.^19^	12.5%	18.2%

### Reconstruction of Time-to-Event Data

**Fig. 3.**
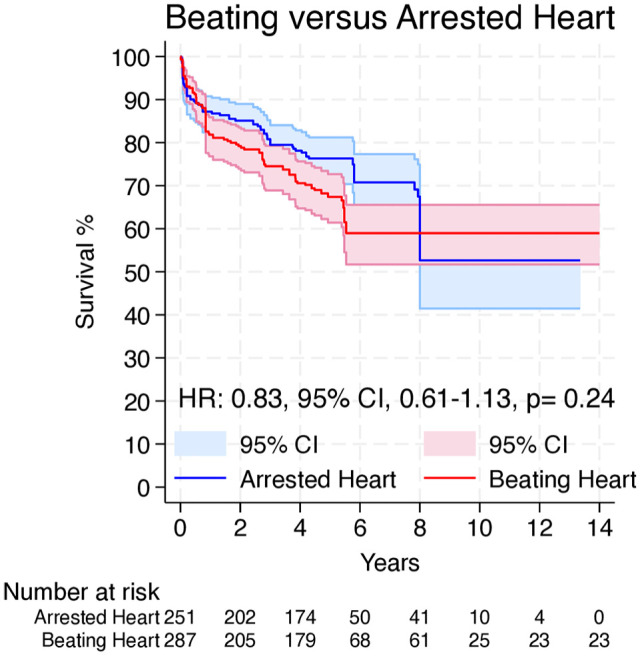
Pooled survival curve for long-term survival. CI, confidence interval; HR, hazard ratio.

Overall, 4 Kaplan–Meier curves were processed, digitalized, and reconstructed. Using the previously described methodology,^13,14^ we extracted the IPD from these curves. The entire observation period was 14 years. Figure 3 shows the pooled curve for long-term survival. There was no significant difference between the 2 strategies in long-term survival (HR = 0.83, 95% CI: 0.61 to 1.13, *P* = 0.24).

### Secondary Outcomes

**Fig. 4.**
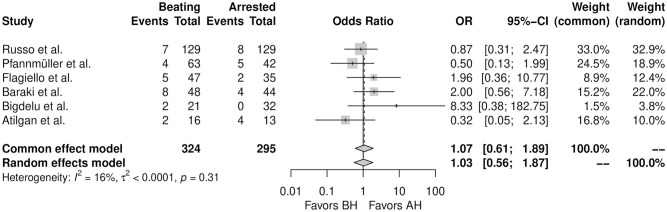
Forest plot for short-term mortality. AH, arrested heart; BH, beating heart; CI, confidence interval; OR, odds ratio.

Table 3 summarizes the main findings. Figure 4 shows the forest plot for short-term survival. There was no significant difference between the 2 strategies regarding short-term survival (OR = 1.03, 95% CI: 0.56 to 1.87, *P* = 0.93). Supplemental Figure 3 shows the forest plot for CPB duration. There was no significant difference between the 2 strategies regarding CPB duration (SMD = −0.61, 95% CI: –1.61 to 0.40, *P* = 0.23). Supplemental Figure 4 shows the forest plot for procedural duration. There was no significant difference between the 2 strategies regarding procedural duration (SMD = 0.31, 95% CI: –0.68 to 1.30, *P* = 54). Supplemental Figure 5 shows the forest plot for ICU LOS. There was no significant difference between the 2 strategies regarding ICU LOS (SMD = −0.44, 95% CI: –1.32 to 0.44, *P* = 0.33). Supplemental Figure 6 shows the forest plot for hospital LOS. There was no significant difference between the 2 strategies regarding hospital LOS (SMD = 0.10, 95% CI: –0.10 to 0.31, *P* = 0.30). Supplemental Figure 7 shows the incidence of perioperative stroke. There was no significant difference between the 2 strategies regarding perioperative stroke (OR = 0.65, 95% CI: 0.10 to 4.14, *P* = 0.65).

**Table 3.** Summary of Outcomes.

Outcome	Number of studies	Number of patients	Effect estimate (95% CI)	*P* value
Permanent pacemaker implantation	4	537	OR = 0.83 (0.45 to 1.51)	0.54
Short-term mortality	6	619	OR = 1.03 (0.56 to 1.87)	0.93
Long-term survival	4	538	HR = 0.83 (0.61 to 1.13)	0.24
CPB duration	6	619	SMD = −0.61 (–1.61 to 0.40)	0.23
Procedural duration	4	279	SMD = 0.31 (–0.68 to 1.30)	0.54
ICU stay	4	256	SMD = −0.44 (–1.32 to 0.44)	0.33
Hospital stay	5	514	SMD = 0.10 (–0.10 to 0.31)	0.30
Perioperative stroke	3	445	OR = 0.65 (0.10 to 4.14)	0.65

Abbreviations: CI, confidence interval; CPB, cardiopulmonary bypass; HR, hazard ratio; ICU, intensive care unit; OR, odds ratio; SMD, standardized mean difference.

## Discussion

We demonstrated in this meta-analysis that performing TV surgery on the BH is not likely to be associated with a reduced risk of postoperative PPI or with different incidences of major clinical endpoints. This finding may be interesting, because the discussion of the potential benefits of a BH approach has been entertained for long.^1,11,12^ Avoiding additional clamp and thereby ischemia times as well as the chance to detect the induction of an AV block by a suture (and the chance to revert it by releasing the suture) are convincing arguments for this approach.^1,11,12^ Nevertheless, we show with our analysis that the discussed advantages cannot be found in the available publications. Thus, the exact surgical technique used may not be of the greatest importance. However, the pacemaker rate following TV surgery is a known problem but has recently been reported as unexpectedly high. The Cardiothoracic Surgical Trials Network tricuspid trial showed that the requirement for PPI after concomitant mitral and tricuspid surgery was 15.2%,^20^ with rates 5 times higher than for isolated mitral surgery. A subsequent analysis of the Mini-Mitral registry (an international registry of patients from 17 expert valve centers) showed a lower rate, but a value of 10% (compared with 5% in isolated mitral cases) is still significant.^21^ The rates of new requirement for PPI in the studies used for our analysis here ranged from 6.3% to 12.5% in the BH group and from 7.1% to 18.2% in the AH group, again hovering around the 2-digit percentage range. This cumulating evidence with repeatedly reported higher rates of pacemaker requirements may suggest that this risk is not directly related to the surgical technique or skill. However, our own data with PPI rates of 0.7% in 136 patients having undergone isolated TV surgery argue against this notion.^22^

Operating on the BH also has the potential to avoid or reduce cardioplegic arrest times. As TV patients often present late in the course of the disease or at advanced age often with signs of liver or kidney impairment, the duration of cardioplegic arrest might become important. We showed in a recent analysis that there is a direct relationship between age, cross-clamp duration, and mortality, thus indicating that, especially in older patients, having shorter cross-clamp times might be beneficial.^23^ Our current work does not support this for isolated TV surgery, which may be due to the fact that procedure times were rather short. That means that this point might still play a role in situations in which TV surgery is performed concomitant to other cardiac surgical procedures. Cross-clamp and even CPB times may then become relevant because the relationship we describe over time follows an exponential pattern with a steep rise in risk after only about 2 h.^23^ The supplemental tables for our previous article show CPB times that were less than 2 h in all studies.

The data we report here were difficult to find. Our initial search strategy revealed only 2 individual reports, and the other 4 reports were identified through further detective work by reviewing the references of other reports. In addition, the patient number in these studies is small, with a total of 767 patients in 6 studies. This observation is in contrast to the reported incidence of TR in the general population and to the current guidelines.^1^ A recent study of all adult residents of Olmsted County, Minnesota, who underwent clinically indicated echocardiography between 1990 and 2000 showed that relevant (greater or equal to moderate) TR is common in community residents and corresponds to a U.S. age-adjusted and sex-adjusted prevalence of 0.55%.^2^ This translates to more than 1.8 million people in the United States affected by greater or equal to moderate TR. Importantly, only 2.6% of patients in this analysis ever had TV surgery during follow-up.^2^

It is difficult to explain this mismatch and even difficult to explain by the available data. Dreyfus et al. just reported a survival advantage of isolated TV surgery as long as the TRISCORE was not high.^6^ In addition, the mortality in the studies of our analysis ranged between 3.8% and 20%, the latter stemming from the smallest report with 6 deaths from 29 patients. The overall mortality was 9.2%, which included a significant fraction of patients with infective endocarditis, in whom expected mortality is often greater than 20%. Considering that the chronic coronary syndrome guideline^24^ defines high risk as expected mortality greater than 8%, it is reasonable to conclude that many patients with an indication for TV surgery are unlikely to be truly high risk. Thus, it may only be speculated what the true reason for this undertreatment could be, but it certainly appears necessary to report these outcomes for better care of patients with TR in the future.

### Study Limitations

This is the first meta-analysis of reconstructed time-to-event data to address this important topic. However, this work has the intrinsic limitations of observational series, including the risk of methodological heterogeneity of the included studies and residual confounders (e.g., center surgical volume, period in which the surgeries were done, and use of minimally invasive approach). Moreover, fluctuations in the survival curves after 6 years may be related to the lack of long follow-up in some of the studies involved in the analysis.

One important factor that could not be considered for subgroup analysis was the distinction between patients with endocarditis and patients with other etiologies. The infected lesion may be the driver for the extent of dissection and potentially for a PPI. Furthermore, the degree of right ventricular failure before and after these surgeries could not be addressed as an isolated approach. Possibly a stratification based on the type of surgery would provide other insights, as the reason for surgery creates totally different physiologic profiles for each patient type. Moreover, most of the studies did not comment about concomitant AF procedures or preexisting bundle branch block or other arrythmias, which is a factor that could affect the PPI rates.

Ultimately, the individual studies did not provide clear information regarding their guidelines for the time between surgery and PPI. The fact that they possibly are based on different thresholds for implantation may vary skew results. Although there was no difference in CPB time between the individual studies and in the overall analysis, a potential evaluation of these values together with cross-clamping time could provide insights into whether cross-clamping time is associated with mortality in these populations, as has been demonstrated in other groups.

## Conclusions

This meta-analysis suggests that performing TV surgery on the BH is not likely to be associated with reduced risk of postoperative PPI or with different incidences of major clinical endpoints. Large sample analysis with randomized design should be conducted to achieve any potential recommendation level in this topic.

## Supplemental Material

sj-pdf-1-inv-10.1177_15569845251351904 – Supplemental material for Beating Versus Arrested Heart Technique for Isolated Tricuspid Valve Surgery: A Meta-Analysis of Reconstructed Time-to-Event Data Supplemental material, sj-pdf-1-inv-10.1177_15569845251351904 for Beating Versus Arrested Heart Technique for Isolated Tricuspid Valve Surgery: A Meta-Analysis of Reconstructed Time-to-Event Data by Tulio Caldonazo, Hristo Kirov, Isabel Niedworok, Angelique Runkel, Johannes Fischer, Murat Mukharyamov and Torsten Doenst in Innovations
