# Supplementary material for: Beating Versus Arrested Heart Technique for Isolated Tricuspid Valve Surgery: A Meta-Analysis of Reconstructed Time-to-Event Data
Source: Innovations (Phila). 2025 Jul 6;20(4):359–66. doi: 10.1177/15569845251351904 (PMC12398632; doi:10.1177/15569845251351904)
Supplement: sj-pdf-1-inv-10.1177_15569845251351904 – Supplemental material for Beating Versus Arrested Heart Technique for Isolated Tricuspid Valve Surgery: A Meta-Analysis of Reconstructed Time-to-Event Data [file sj-pdf-1-inv-10.1177_15569845251351904.pdf]

Supplemental Table 1. Search Strategy for Ovid MEDLINE.

((tricuspid valve) OR (tricuspid valve repair) OR (tricuspid valve reconstruction) OR (tricuspid valve replacement) OR (tricuspid valve surgery) OR (tricuspid valve anuloplasty)) AND ((Beating heart) OR (arrested heart) OR (fibrillating heart)) AND ((pacemaker implantations) OR (pacemaker) OR (complications) OR (postoperative complications))

("tricuspid valve"[MeSH Terms] OR ("tricuspid"[All Fields] AND "valve"[All Fields]) OR "tricuspid valve"[All Fields] OR (("tricuspid valve"[MeSH Terms] OR ("tricuspid"[All Fields] AND "valve"[All Fields]) OR "tricuspid valve"[All Fields]) AND ("repairability"[All Fields] OR "repairable"[All Fields] OR "repaire"[All Fields] OR "repaired"[All Fields] OR "repairment"[All Fields] OR "wound healing"[MeSH Terms] OR ("wound"[All Fields] AND "healing"[All Fields]) OR "wound healing"[All Fields] OR "repair"[All Fields] OR "repairing"[All Fields] OR "repairs"[All Fields])) OR (("tricuspid valve"[MeSH Terms] OR ("tricuspid"[All Fields] AND "valve"[All Fields]) OR "tricuspid valve"[All Fields]) AND ("plastic surgery procedures"[MeSH Terms] OR ("plastic"[All Fields] AND "surgery"[All Fields] AND "procedures"[All Fields]) OR "plastic surgery procedures"[All Fields] OR "reconstruction"[All Fields] OR "reconstructions"[All Fields] OR "reconstruct"[All Fields] OR "reconstructability"[All Fields] OR "reconstructable"[All Fields] OR "reconstructed"[All Fields] OR "reconstructible"[All Fields] OR "reconstructing"[All Fields] OR "reconstructional"[All Fields] OR "reconstructive"[All Fields] OR "reconstructs"[All Fields])) OR (("tricuspid valve"[MeSH Terms] OR ("tricuspid"[All Fields] AND "valve"[All Fields]) OR "tricuspid valve"[All Fields]) AND ("replace"[All Fields] OR "replaceable"[All Fields] OR "replaced"[All Fields] OR "replaces"[All Fields] OR "replacing"[All Fields] OR "replacment"[All Fields] OR "replantation"[MeSH Terms] OR "replantation"[All Fields] OR "replacement"[All Fields] OR "replacements"[All Fields])) OR (("tricuspid valve"[MeSH Terms] OR ("tricuspid"[All Fields] AND "valve"[All Fields]) OR "tricuspid valve"[All Fields]) AND ("surgery"[MeSH Subheading] OR "surgery"[All Fields] OR "surgical procedures, operative"[MeSH Terms] OR ("surgical"[All Fields] AND "procedures"[All Fields] AND "operative"[All Fields]) OR "operative surgical procedures"[All Fields] OR "general surgery"[MeSH Terms] OR ("general"[All Fields] AND "surgery"[All Fields]) OR "general surgery"[All Fields] OR "surgery s"[All Fields] OR "surgerys"[All Fields] OR "surgeries"[All Fields])) OR (("tricuspid valve"[MeSH Terms] OR ("tricuspid"[All Fields] AND "valve"[All Fields]) OR "tricuspid valve"[All Fields]) AND "anuloplasty"[All Fields])) AND ((("beating"[All Fields] OR "beatings"[All Fields] OR "beats"[All Fields]) AND ("heart"[MeSH Terms] OR "heart"[All Fields] OR "hearts"[All Fields] OR "heart s"[All Fields])) OR (("arrestant"[All Fields] OR "arrestants"[All Fields] OR "arresting"[All Fields] OR "arrestment"[All Fields] OR "arrests"[All Fields] OR "heart arrest"[MeSH Terms] OR ("heart"[All Fields] AND "arrest"[All Fields]) OR "heart arrest"[All Fields] OR "arrest"[All Fields] OR "arrested"[All Fields]) AND ("heart"[MeSH Terms] OR "heart"[All Fields] OR "hearts"[All Fields] OR "heart s"[All Fields])) OR (("fibril"[All Fields] OR "fibril s"[All Fields] OR "fibrilation"[All Fields] OR "fibrilization"[All Fields] OR "fibrilized"[All Fields] OR "fibrillate"[All Fields] OR "fibrillated"[All Fields] OR "fibrillates"[All Fields] OR "fibrillating"[All Fields] OR "fibrillation"[All Fields] OR "fibrillations"[All Fields] OR "fibrillization"[All Fields] OR "fibrillize"[All Fields] OR "fibrillized"[All Fields] OR "fibrillizes"[All Fields] OR "fibrillizing"[All Fields] OR "fibrillous"[All Fields] OR "fibrills"[All Fields] OR "fibrils"[All Fields]) AND ("heart"[MeSH Terms] OR "heart"[All Fields] OR "hearts"[All Fields] OR "heart s"[All Fields]))) AND ((("pacemaker s"[All Fields] OR "pacemaker, artificial"[MeSH Terms] OR ("pacemaker"[All Fields] AND "artificial"[All Fields]) OR "artificial pacemaker"[All Fields] OR "pacemaker"[All Fields] OR "pacemakers"[All Fields] OR "pacemaking"[All Fields]) AND ("drug implants"[MeSH Terms] OR ("drug"[All Fields] AND "implants"[All Fields]) OR "drug implants"[All Fields] OR "implant"[All Fields] OR "embryo implantation"[MeSH Terms] OR ("embryo"[All Fields] AND "implantation"[All Fields]) OR "embryo implantation"[All Fields] OR "implantation"[All Fields] OR "implant s"[All Fields] OR "implantability"[All Fields] OR "implantable"[All Fields] OR "implantables"[All Fields] OR "implantate"[All Fields] OR "implantated"[All Fields] OR "implantates"[All Fields] OR "implantations"[All Fields] OR "implanted"[All Fields] OR "implanter"[All Fields] OR "implanters"[All Fields] OR "implanting"[All Fields] OR

"implantion"[All Fields] OR "implantitis"[All Fields] OR "implants"[All Fields])) OR ("pacemaker s"[All Fields] OR "pacemaker, artificial"[MeSH Terms] OR ("pacemaker"[All Fields] AND "artificial"[All Fields]) OR "artificial pacemaker"[All Fields] OR "pacemaker"[All Fields] OR "pacemakers"[All Fields] OR "pacemaking"[All Fields]) OR ("complicances"[All Fields] OR "complicate"[All Fields] OR "complicated"[All Fields] OR "complicates"[All Fields] OR "complicating"[All Fields] OR "complication"[All Fields] OR "complication s"[All Fields] OR "complications"[MeSH Subheading] OR "complications"[All Fields]) OR ("postoperative complications"[MeSH Terms] OR ("postoperative"[All Fields] AND "complications"[All Fields]) OR "postoperative complications"[All Fields]))
**Translations**
tricuspid valve: "tricuspid valve"[MeSH Terms] OR ("tricuspid"[All Fields] AND "valve"[All Fields]) OR "tricuspid valve"[All Fields]<br>tricuspid valve: "tricuspid valve"[MeSH Terms] OR ("tricuspid"[All Fields] AND "valve"[All Fields]) OR "tricuspid valve"[All Fields]<br>repair: "repairability"[All Fields] OR "repairable"[All Fields] OR "repaire"[All Fields] OR "repaired"[All Fields] OR "repairment"[All Fields] OR "wound healing"[MeSH Terms] OR ("wound"[All Fields] AND "healing"[All Fields]) OR "wound healing"[All Fields] OR "repair"[All Fields] OR "repairing"[All Fields] OR "repairs"[All Fields]<br>tricuspid valve: "tricuspid valve"[MeSH Terms] OR ("tricuspid"[All Fields] AND "valve"[All Fields]) OR "tricuspid valve"[All Fields]<br>reconstruction: "plastic surgery procedures"[MeSH Terms] OR ("plastic"[All Fields] AND "surgery"[All Fields] AND "procedures"[All Fields]) OR "plastic surgery procedures"[All Fields] OR "reconstruction"[All Fields] OR "reconstructions"[All Fields] OR "reconstruct"[All Fields] OR "reconstructability"[All Fields] OR "reconstructable"[All Fields] OR "reconstructed"[All Fields] OR "reconstructible"[All Fields] OR "reconstructing"[All Fields] OR "reconstructional"[All Fields] OR "reconstructive"[All Fields] OR "reconstructs"[All Fields]<br>tricuspid valve: "tricuspid valve"[MeSH Terms] OR ("tricuspid"[All Fields] AND "valve"[All Fields]) OR "tricuspid valve"[All Fields]<br>replacement: "replace"[All Fields] OR "replaceable"[All Fields] OR "replaced"[All Fields] OR "replaces"[All Fields] OR "replacing"[All Fields] OR "replacment"[All Fields] OR "replantation"[MeSH Terms] OR "replantation"[All Fields] OR "replacement"[All Fields] OR "replacements"[All Fields]<br>tricuspid valve: "tricuspid valve"[MeSH Terms] OR ("tricuspid"[All Fields] AND "valve"[All Fields]) OR "tricuspid valve"[All Fields]<br>surgery: "surgery"[Subheading] OR "surgery"[All Fields] OR "surgical procedures, operative"[MeSH Terms] OR ("surgical"[All Fields] AND "procedures"[All Fields] AND "operative"[All Fields]) OR "operative surgical procedures"[All Fields] OR "general surgery"[MeSH Terms] OR ("general"[All Fields] AND "surgery"[All Fields]) OR "general surgery"[All Fields] OR "surgery's"[All Fields] OR "surgerys"[All Fields] OR "surgeries"[All Fields]<br>tricuspid valve: "tricuspid valve"[MeSH Terms] OR ("tricuspid"[All Fields] AND "valve"[All Fields]) OR "tricuspid valve"[All Fields]<br>Beating: "beating"[All Fields] OR "beatings"[All Fields] OR "beats"[All Fields]<br>heart: "heart"[MeSH Terms] OR "heart"[All Fields] OR "hearts"[All Fields] OR "heart's"[All Fields]<br>arrested: "arrestant"[All Fields] OR "arrestants"[All Fields] OR "arresting"[All Fields] OR "arrestment"[All Fields] OR "arrests"[All Fields] OR "heart arrest"[MeSH Terms] OR ("heart"[All Fields] AND "arrest"[All Fields]) OR "heart arrest"[All Fields] OR "arrest"[All Fields] OR "arrested"[All Fields]<br>heart: "heart"[MeSH Terms] OR "heart"[All Fields] OR "hearts"[All Fields] OR "heart's"[All Fields]<br>fibrillating: "fibril"[All Fields] OR "fibril's"[All Fields] OR "fibrilation"[All Fields] OR "fibrilization"[All Fields] OR "fibrilized"[All Fields] OR "fibrillate"[All Fields] OR "fibrillated"[All Fields] OR "fibrillates"[All Fields] OR "fibrillating"[All Fields] OR "fibrillation"[All Fields] OR "fibrillations"[All Fields] OR "fibrillization"[All Fields] OR "fibrillize"[All Fields] OR "fibrillized"[All Fields] OR "fibrillizes"[All Fields] OR "fibrillizing"[All

Fields] OR "fibrillous"[All Fields] OR "fibrills"[All Fields] OR "fibrils"[All Fields]
heart: "heart"[MeSH Terms] OR "heart"[All Fields] OR "hearts"[All Fields] OR "heart's"[All Fields]
pacemaker: "pacemaker's"[All Fields] OR "pacemaker, artificial"[MeSH Terms] OR ("pacemaker"[All Fields] AND "artificial"[All Fields]) OR "artificial pacemaker"[All Fields] OR "pacemaker"[All Fields] OR "pacemakers"[All Fields] OR "pacemaking"[All Fields]
implantations: "drug implants"[MeSH Terms] OR ("drug"[All Fields] AND "implants"[All Fields]) OR "drug implants"[All Fields] OR "implant"[All Fields] OR "embryo implantation"[MeSH Terms] OR ("embryo"[All Fields] AND "implantation"[All Fields]) OR "embryo implantation"[All Fields] OR "implantation"[All Fields] OR "implant's"[All Fields] OR "implantability"[All Fields] OR "implantable"[All Fields] OR "implantables"[All Fields] OR "implantate"[All Fields] OR "implantated"[All Fields] OR "implantates"[All Fields] OR "implantations"[All Fields] OR "implanted"[All Fields] OR "implanter"[All Fields] OR "implanters"[All Fields] OR "implanting"[All Fields] OR "implantion"[All Fields] OR "implantitis"[All Fields] OR "implants"[All Fields]
pacemaker: "pacemaker's"[All Fields] OR "pacemaker, artificial"[MeSH Terms] OR ("pacemaker"[All Fields] AND "artificial"[All Fields]) OR "artificial pacemaker"[All Fields] OR "pacemaker"[All Fields] OR "pacemakers"[All Fields] OR "pacemaking"[All Fields]
complications: "complicances"[All Fields] OR "complicate"[All Fields] OR "complicated"[All Fields] OR "complicates"[All Fields] OR "complicating"[All Fields] OR "complication"[All Fields] OR "complication's"[All Fields] OR "complications"[Subheading] OR "complications"[All Fields]
postoperative complications: "postoperative complications"[MeSH Terms] OR ("postoperative"[All Fields] AND "complications"[All Fields]) OR "postoperative complications"[All Fields]

Supplemental Table 2. Newcastle-Ottawa Risk Of Bias Assessment Scale.

Study	Selection	Comparability	Outcome/exposure
Bigdelu et al. [1]	****	*	**
Russo et al. [2]	****	**	***
Flagiello et al. [3]	****	**	**
Atılgan et al. [4]	***	*	**
Baraki et al. [5]	****	*	**
Pfannmüller et al. [6]	****	*	***

Supplemental Table 3. Preoperative and Perioperative Data of Included Patients From the Selected Studies.

**Study**	**Age, years**		**Female**		**LVEF, %**		**HTN**		**DM**		**COPD**		**AF**		**Prior cardiac surgery**	
	**BH**	**AH**	**BH**	**AH**	**BH**	**AH**	**BH**	**AH**	**BH**	**AH**	**BH**	**AH**	**BH**	**AH**	**BH**	**AH**
Bigdelu et al. [1]	49.5±52.9	49.7±52.2	76.2	25.0	39.8±9.5	38.1±1.4	38.1	37.5	37.5	15.6	19.0	12.5	76.2	68.8	38.1	84.4
Russo et al. [2]	58.0±15.0	56.0±15.0	59.7	56.6	NR	NR	NR	NR	18.	14.7	10.9	14.7	NR	NR	55.8	41.1
Flagiello et al. [3]	61.8±16.6	55.4±15.8	55.3	57.1	59.6±.0	58.0±8.8	63.8	31.4	8.5	14.3	10.6	14.3	44.7	37.1	53.2	28.6
Atılgan et al. [4]	57.9±8.4	55.4±1.6	62.5	61.5	58.7±8.0	57.2±7.6	NR	NR	NR	NR	NR	NR	68.7	69.2	75.0	61.5
Baraki et al. [5]	53.6±19.5	54.3±19.6	50.0	54.5	NR	NR	NR	NR	6.3	2.3	10.5	4.5	29.2	29.5	66.7	22.7
Pfannmüller et al. [6]	65.2±11.9	55.2±17.3	66.7	45.2	56.6±10.3	61.2±11.8	57.1	52.4	20.6	14.3	NR	NR	42.9	14.3	68.3	26.2

Abbreviations: AF, atrial fibrillation; AH, arrested heart; BH, beating heart; COPD, chronic obstructive pulmonary disease; DM, diabetes mellitus; HTN, hypertension; LVEF, left ventricular ejection fraction; NR, not reported.

Data are reported as mean ± standard deviation or percentage.

Supplemental Table 4. Preoperative and Perioperative Data of Included Patients From the Selected Studies.

Study	Preexisting permanent pacemaker		Ascites		Chronic renal failure		Endocarditis indication		Moderate/severe TR		TV repair		Median sternotomy		Urgent/emergent surgery	
	**BH**	**AH**	**BH**	**AH**	**BH**	**AH**	**BH**	**AH**	**BH**	**AH**	**BH**	**AH**	**BH**	**AH**	**BH**	**AH**
Bigdelu et al. [1]	NR	NR	57.1	57.1	NR	NR	NR	NR	81.0	68.8	23.8	34.4	76.2	100.0	NR	NR
Russo et al. [2]	NR	NR	NR	NR	NR	NR	14.7	23.3	95.3	95.3	41.9	50.4	79.1	83.7	20.2	21.7
Flagiello et al. [3]	14.9	17.1	NR	NR	36.2	20.0	12.8	25.7	NR	NR	14.9	20.0	100.0	100.0	12.8	22.9
Atılgan et al. [4]	NR	NR	NR	NR	NR	NR	NR	NR	NR	NR	0.0	0.0	43.7	61.5	NR	NR
Baraki et al. [5]	NR	NR	22.9	9.1	43.8	13.6	25.0	38.6	NR	NR	33.3	40.9	75.0	81.8	20.8	4.5
Pfannmüller et al. [6]	42.9	39.5	NR	NR	NR	NR	15.9	57.1	86.9	48.6	81.0	59.5	19.0	61.9	NR	NR

Abbreviations: AH, arrested heart; BH, beating heart; NR, not reported; TR, tricuspid regurgitation; TV, tricuspid valve.

Data are reported as percentage.

Supplemental Figure 1. Leave-one-out analysis for the primary outcome of permanent pacemaker implantation. CI, confidence interval; OR, odds ratio.

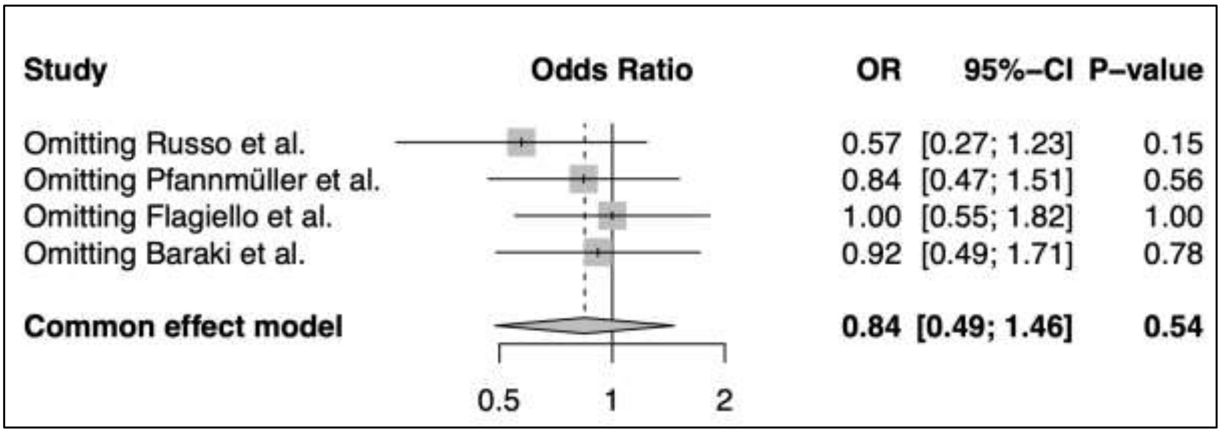

Supplemental Figure 2. Funnel plot for the primary outcome of permanent pacemaker implantation.

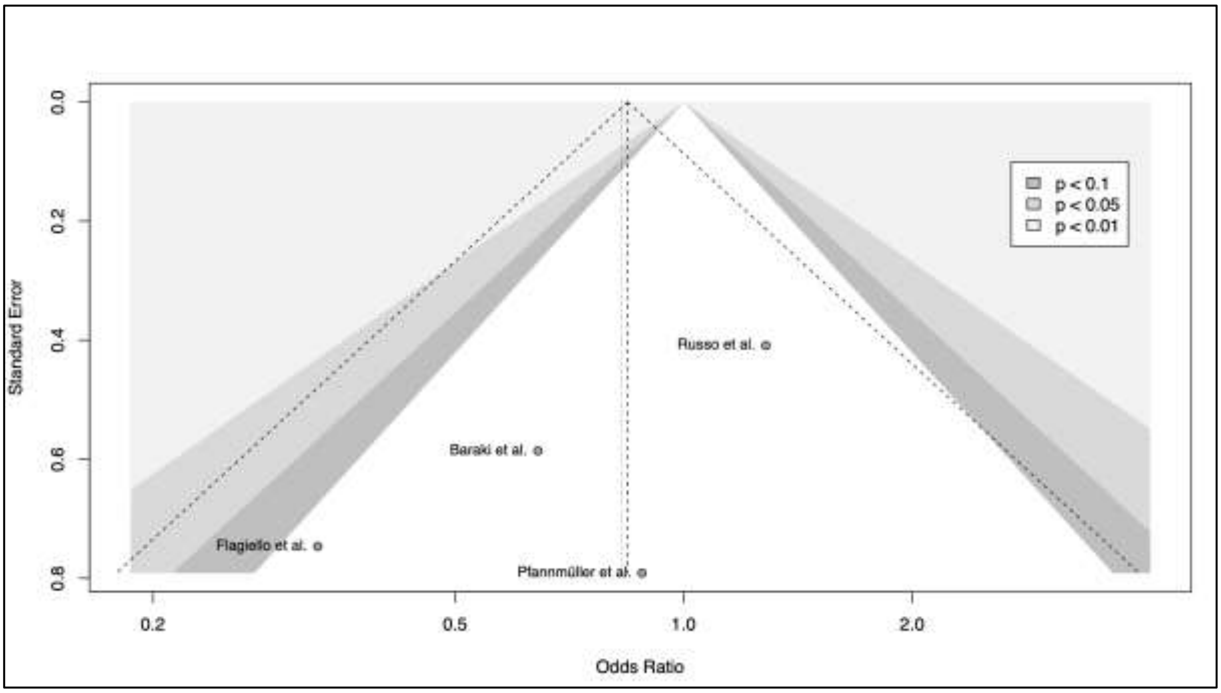

Supplemental Figure 3. Forest plot for cardiopulmonary bypass duration. AH, arrested heart; BH, beating heart; CI, confidence interval; SD, standard deviation; SMD, standardized mean difference.

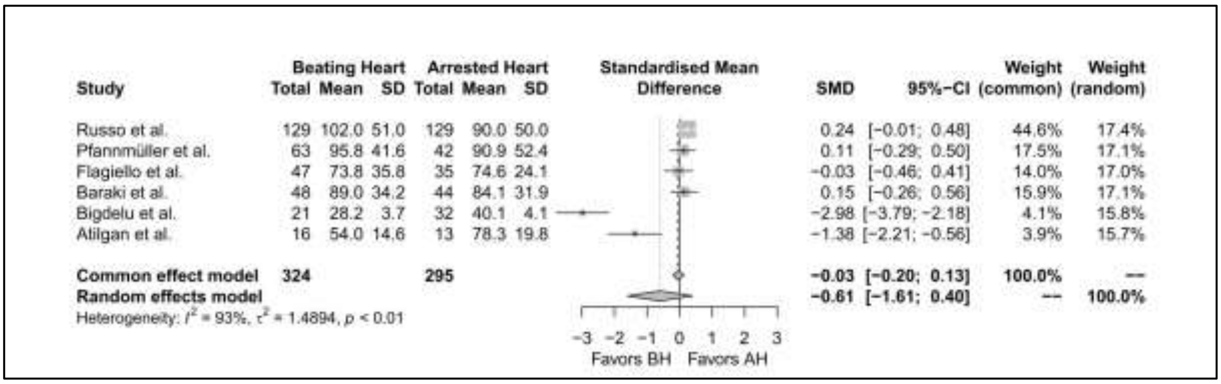

Supplemental Figure 4. Forest plot for procedural duration. AH, arrested heart; BH, beating heart; CI, confidence interval; SD, standard deviation; SMD, standardized mean difference.

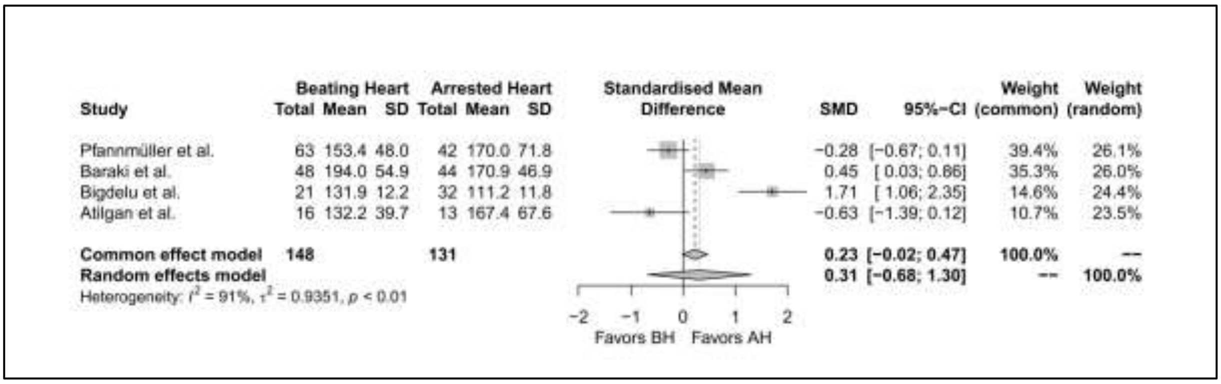

Supplemental Figure 5. Forest plot for intensive care unit length of stay. AH, arrested heart; BH, beating heart; CI, confidence interval; SD, standard deviation; SMD, standardized mean difference.

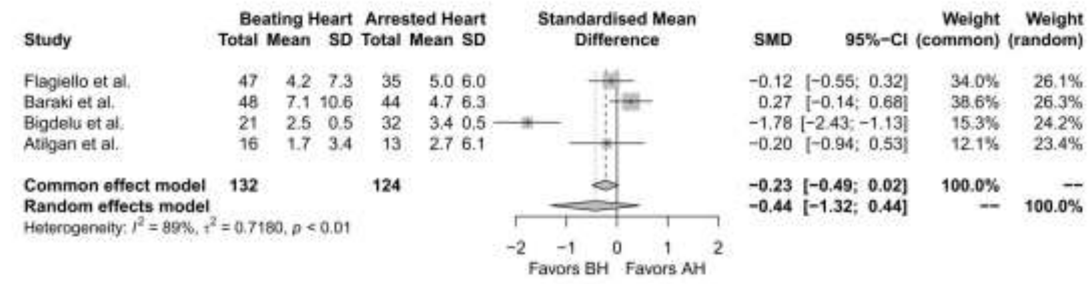

Supplemental Figure 6. Forest plot for hospital length of stay. AH, arrested heart; BH, beating heart; CI, confidence interval; SD, standard deviation; SMD, standardized mean difference.

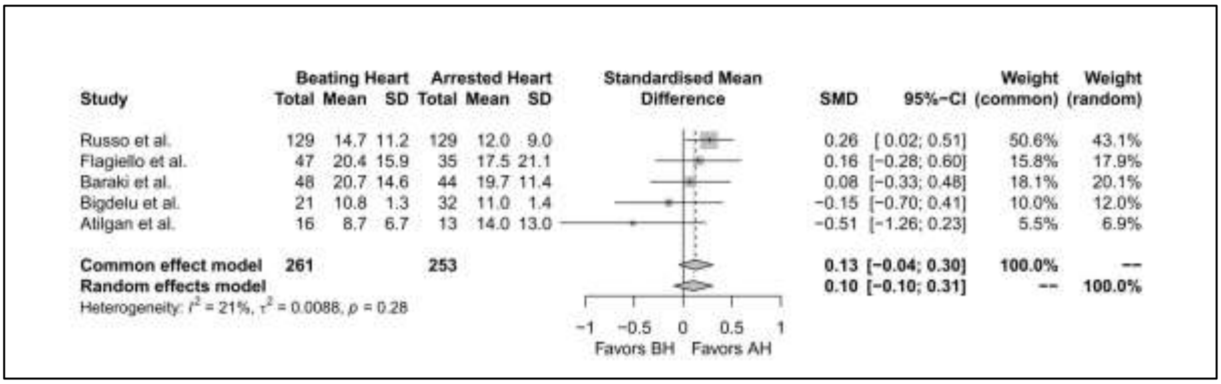

Supplemental Figure 7. Forest plot for perioperative stroke. AH, arrested heart; BH, beating heart; CI, confidence interval; OR, odds ratio.

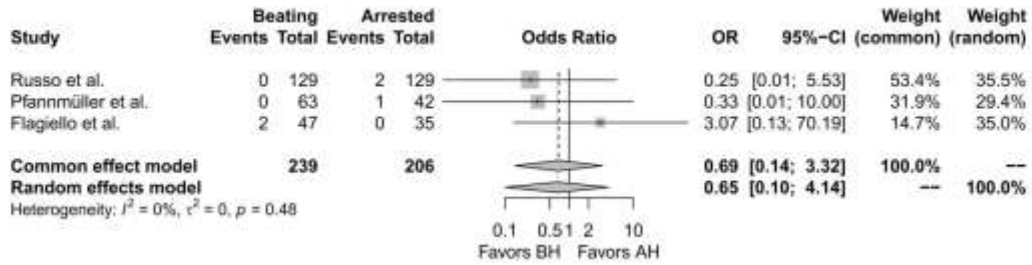

Supplemental References

1. Bigdelu L, Azari A, Mashayekhi Z, et al. A comparative study on the results of beating and arrested heart isolated tricuspid valve surgery: a cross-sectional study. *Health Sci Rep* 2022; 5: e702.

2. Russo M, Di Mauro M, Saitto G, et al. Beating versus arrested heart isolated tricuspid valve surgery: long-term outcomes. *Ann Thorac Surg* 2022; 113: 585–592.

3. Flagiello M, Grinberg D, Connock M, et al. Beating versus arrested heart isolated tricuspid valve surgery: an 11-year experience in the current era. *J Card Surg* 2021; 36: 1020–1027.

4. Atılgan K and Demirdaş E. Beating heart technique in tricuspid valve replacement among patients which have a TAPSE index lower than 15 mm. *J Surg Med* 2020; 4: 212–216.

5. Baraki H, Saito S, Al Ahmad A, et al. Beating heart versus arrested heart isolated tricuspid valve surgery. *Int Heart J* 2015; 56: 400–407.

6. Pfannmuller B, Davierwala P, Misfeld M, et al. Postoperative outcome of isolated tricuspid valve operation using arrested-heart or beating-heart technique. *Ann Thorac Surg* 2012; 94: 1218–1222.
